# *Lactobacillus plantarum* GX17 benefits growth performance and improves functions of intestinal barrier/intestinal flora among yellow-feathered broilers

**DOI:** 10.3389/fimmu.2023.1195382

**Published:** 2023-07-03

**Authors:** Yangyan Yin, Yuying Liao, Jun Li, Zhe Pei, Leping Wang, Yan Shi, Hongyan Peng, Yizhou Tan, Changting Li, Huili Bai, Chunxia Ma, Yu Gong, Tianchao Wei, Hao Peng

**Affiliations:** ^1^ Guangxi Key Laboratory of Veterinary Biotechnology, Guangxi Veterinary Research Institute, Nanning, China; ^2^ Key Laboratory of China(Guangxi)-Association of Southeast Asian Nations (ASEAN) Cross-border Animal Disease Prevention and Control, Ministry of Agriculture and Rural Affairs of China, Nanning, China; ^3^ Institute of Animal Science and Technology, Guangxi University, Nanning, China; ^4^ Virginia Tech, Department of Engineering, Blacksburg, New York, NY, United States; ^5^ Guizhou Provincial Livestock and Poultry Genetic Resources Management Station, Guiyang, China

**Keywords:** *Lactobacillus plantarum*, yellow-feather broiler, growth performance, immunity, intestinal flora population

## Abstract

*Lactobacillus plantarum* has recently been found to be a natural source feed additive bacteria with great advantages in food safety and animal welfare. Discovering novel strains with commercial application potentiation could benefit the local poultry industry, and in particular support Chinese farmers. In this study, we tested a recently isolated novel strain of *Lactobacillus plantarum* GX17 as a feed additive on the growth performance and intestinal barrier functions of 1-day-old Chinese yellow-feather chicks. As good as other commercial probiotics, feeding with *Lactobacillus plantarum* GX17 showed significant improvements in humoral immune responses and enhanced the immune effect after vaccination for either the Newcastle disease vaccine or the avian influenza vaccine. This study also found that feeding with *Lactobacillus plantarum* GX17 improved the feed-to-weight ratio and caused a significant increase of the villus length to crypt depth ratio. Furthermore, *Lactobacillus plantarum* GX17 significantly up-regulated the mRNA expression of CLDN, MUC2, and TLR2, all of which are jejunum-associated barrier genes, indicating an improvement of the intestinal barrier functions by enhancing the tight junction between epithelia cells. These results are comparable to the effects of feeding the commercial complex probiotics that improve the expression levels of CLDN, ocludin, MUC2, TLR2, and TLR4. In terms of maintaining intestinal health, commercial complex probiotics increased the relative abundance of *Parabacteroides* and *Romboutsia*, while *Lactobacillus plantarum* GX17 increased the relative abundance of *Pseudoflavonifractor*. Our data suggest that *Lactobacillus plantarum* GX17 could enhance the intestinal absorption of nutrients and therefore improve the growth performance of Chinese yellow-feather chicks. In conclusion, compared with the commercial complex probiotics, *Lactobacillus plantarum* GX17 has more positive effects on the growth performance and intestinal barrier function of yellow-feather chickens, and can be used as a feed additive.

## Introduction

Nowadays, the speed with which pathogens can develop tolerance to antibiotics can be even faster than the drug discovery of new anti-microbial compounds. It has become a great challenge for public health globally. Antibiotic residues in animal products and antibiotic abuse caused the emergence of resistant strains and are the two main reasons for the evolution of pathogens. China and the European Union banned the use of growth-promoting antibiotics as feed additives in 2020 and 2006, respectively (1), which helped to control the spread of antibiotic-resistant strains (2). However, avoiding antibiotic use in feed has introduced new challenges, including maintaining growth performance and suppressing bacterial infections in broilers (3).. Therefore, it has become crucial to develop safe and reliable alternatives to antibiotics in the poultry industry. Probiotics have been extensively studied as a potential alternative to antibiotic growth promoters, and for the search for qualified bacterial strains has attracted lots of attention recently in the field.

With positive effects in disease prevention and control as well as growth promotion, probiotics have been widely used in livestock and poultry industries (4–8). *Lactobacillus plantarum* is one of the most commonly used lactic acid bacteria. It has positive probiotic effects and can improve the growth performance of broiler chickens (9–11) and had good effects in the prevention and control of diseases (12). For example, adding optimized amounts of *Lactobacillus plantarum* to feed increased the average daily intake, body weight, dry matter, nitrogen, and total energy of broilers in terms of whole intestinal apparent digestibility, significantly reduced the amount of *E. coli*, and increased the number of lactic acid bacteria (13). In addition, *Lactobacillus plantarum* exhibited antibacterial activity against *Salmonella in vitro*: it significantly inhibited the growth, adhesion, and invasion of *Salmonella*, while greatly reducing their biofilm formation capacity (14). Also, feeding of *Lactobacillus plantarum* could reduce the number of *Salmonella typhimurium* and slow down the inflammatory responses to infection in neonatal broilers by modulating miRNA expression (15). However, the safety of probiotics is under debate and needs to be further explored. It has been reported that taking probiotic supplementation among seniors over 69 years of age leads not only to an increase in potentially beneficial gut bacteria, but also to an increase in activation of nonspecific immune responses (16). While immune-compromised hosts may benefit most from probiotic supplementation, these groups may also be at higher risk for adverse effects, such as the development of sepsis, due to their reduced ability to clear microorganisms (17). Although probiotics are commonly reported to protect the intestinal barrier, there may be cases where probiotics not only fail to restore the intestinal barrier but also promote translocation or self-induced infections (18). Meanwhile, the use of *Lactobacillus plantarum* as an additive in drinking water for chickens of native Chinese yellow-feathered broilers has not been studied. Yellow-feather broiler chickens have a smaller body weight, longer growth cycle, and tasty meat quality, which makes them suitable for use in cooking. Therefore, compared to white feather broilers (mainly sold to KFC, McDonald’s, and other fast-food restaurant chains), yellow-feather broilers are more popular among Chinese consumers with a market of 4.43 billion broilers during 2020 (19). As yellow-feather broilers are usually raised in free-range farms, and the breeding time is two to three times longer than that of white-feather broilers, they are more susceptible to infection by harmful bacteria (20), and the demand for alternative antibacterial products is more urgent compared to large-scale farmed white-feather broilers.

A novel strain of *Lactobacillus plantarum* GX17 has been isolated and identified in our laboratory and was screened *in vitro* for its good antibacterial effects, but its role as a feed additive on broiler growth performance and barrier function is unclear. This study aimed to compare the growth performance and health status of broiler chickens when feeding them *Lactobacillus plantarum* GX17 or commercial complex probiotics diet. The aim was also to explore the cooperation between single probiotic and commercial complex probiotic to investigate the potential of *Lactobacillus plantarum* GX17 in livestock and poultry production applications. This study was designed for and conducted over a 42-day feeding trial. The effects of *Lactobacillus plantarum* GX17 on growth parameters, immunological indices, jejunal intestinal morphology, jejunal barrier function, and cecum intestinal flora of broilers were investigated. This study provides theoretical data for the application effects of *Lactobacillus plantarum* GX17 on the quality and productivity of yellow-feathered broiler chickens.

## Materials and method

### Sources of materials

All animal procedures were performed in accordance with the protocols approved by the Institutional Animal Experimental Ethical Inspection Form of Guangxi Veterinary Research Institute, Nanning, China(8/2014/JU). The probiotic, *Lactobacillus plantarum* GX17, with a final feeding concentration of 1.5 × 10~9 CFU/animal, was kept by the Key Laboratory of Veterinary Biotechnology of Guangxi Veterinary Research Institute, Guangxi, China. The commercial compound probiotic reagent was purchased from a local biological company (Shandong Baolaililai Biological Engineering Co., Ltd.).

### Experimental design and animal husbandry

A total of 225 1-day-old yellow-feathered chicks were randomly divided into three groups(15×5 animals per group) with similar body weight: the *Lactobacillus plantarum* GX17 feeding group(LP-group), the commercial complex probiotics feeding group (S-group), and the control group (Group-C). Group C was fed the basal diet without probiotics; group LP was fed the basal diet with *Lactobacillus plantarum* GX17 (1.5×10~9 CFU/each) in the drinking water; group S was fed the basal diet and drinking water with commercial compound probiotics (1.5×10~9 CFU/each). The test diets were formulated in accordance with the Chicken Feeding Standard (Chinese National Standard Protocol: NY/T33-2004) which combined the growth cycle and breed characteristics of yellow-feathered broiler chickens. **Table 1** summarizes the composition and nutritional levels of these diets (all diets were fortified without antibiotics). Yellow-feathered broilers were maintained at an initial temperature of 31°C and gradually reduced by 2°C/week till 25°C by the end of the 3rd week. Body weight (BW) and feed intake (FI) of all test animals were recorded on the last day of the experiment.

**Table 1:** Composition of basal diets (air-dry basis).

Items	1 to 21 days (%)	22 to 42 days (%)
Corn	55.00	59.00
Soybean meal	36.20	32.40
Soybean oil	3.90	3.90
Limestone	1.15	1.15
NaCl	0.25	0.25
CaHPO4	1.95	1.75
Premix^1(2)^	1.55	1.55
Total	100.00	100.00
Nutrient levels		
ME(MJ/kg) ^3)^	12.45	12.15
CP	21.15	19.75
Ca	0.92	0.84
P	0.65	0.58
Lysine	1.18	0.87
Methionine	0.47	0.38

^1)^1–21 The premix for 1 to 21 days of ages provided the following per kg of diets:VA 15000 IU, VD 35100 IU, VE 19.2 IU, VK_3_ 2.4 mg, VB_1_ 1.2 mg, VB_2_ 10.2 mg, VB_6_ 2.4 mg, VB_12_ 0.012 mg, D-pantothenic acid12 mg, nicotinic acid 39 mg, folic acid 1.2 mg, biotin 0.189 mg, choline 700 mg, Gu (as copper sulfate)8 mg, Mn (as manganese sulfate) 100 mg, Fe (as ferrous sulfate) 80 mg, Zn (as zinc sulfate) 60 mg, I (as potassium iodide)0.35 mg, and Se (as sodium selenite) 0.15 mg;

^2)^22–42 The premix for 22 to 42 days of ages provided the following per kg of diets:VA 10000 IU, VD33400 IU, VE 12.8 IU, VK_3_ 1.6 mg, VB_1_ 0.8 mg, VB_2_ 68 mg, VB_6_ 1.6 mg, VB_12_ 0.008 mg, D-pantothenic acid 8 mg, nicotinic acid 26 mg, folic acid 0.8 mg, biotin 0.126 mg, choline 500 mg, Gu (as copper sulfate)8 mg, Mn (as manganese sulfate) 100 mg, Fe (as ferrous sulfate) 80 mg, Zn (as zinc sulfate) 60 mg, I (as potassium iodide)0.35 mg, and Se (as sodium selenite) 0.15 mg;

^3)^ ME is calculated value. Other nutrient levels are measured values.

### The evaluation of growth performances

The status, appetite, and defecation of all testing chicks were observed and recorded daily. Each group of chicks was weighed on the last day of the experiment and fasted for 12 h without water before weighing. The amount of feed supplied, the amount of remaining feed, and the amount of lost feed at the last stage were recorded. The initial weight, final weight, feed intake, and leftover amount of each stage were accurately recorded during the experiment, and the average daily gain (ADG), average daily feed intake (ADFI), and feed conversion ratio (FCR) were calculated. The following formula were used in this study: Average daily weight gain = (average weight at the end of the trial - average weight at the beginning)/number of days in the trial; Average daily feed intake = total feed consumption during the test period/(number of test days × number of chickens); and Feed conversion ratio = average daily feed intake/average daily weight gain.

### The determination of immune organ indexes

On day 21 and day 42 of the experiment, three broilers were randomly selected per replicate to be weighed and sacrificed. The spleen, thymus, and bursa were carefully removed, isolating the adipose tissue, each of the immune organs were weighed and calculated for the immune organ index. The immune organ indexes were generally the weight of the organ (mg) per 10 g body weight using the following formulas: Thymus index = [thymus weight (g) * 10^3^]/[live chicken weight (g)/10] = [thymus weight (g)/live chicken weight (g)] * 10^4^. Spleen index = [spleen weight (g) * 10^3^]/[live chicken weight (g)/10] = [spleen weight (g)/live chicken weight (g)] * 10^4^. Bursal index = [bursal weight(g)* 10^3^]/[live chicken weight(g)/10] = [bursal weight(g)/live chicken weight(g)]*10^4^.

### Immuno-antibody assay

Immunization procedures were comprised of immunization by subcutaneous injection of MD vaccine at 1 day of age, 7-day-old eye-intranasal immunization with new branch combined vaccine (LaSota strain + H120 strain), and 14-day-old new tributary triple vaccine 0.5mL subcutaneous injection (LaSota strain + M41 strain + H2 strain). On the 7^th^ and 28^th^ day after immunization, three animals were randomly selected per replicate. Blood was collected from wind veins and serum was isolated and tested for the potency of antibodies for Newcastle disease and avian influenza H9. The hemagglutination inhibition assay was used to titrate the antibody responses.

### Measurement of serum immunological indicators

Three chickens were randomly selected per replicate on day 21 and day 42 and fasted for 8 hours, after which 5 mL of blood was collected from the wing vein of each animal using a non-anticoagulated blood collection tube. Whole blood samples sat for 30 min at room temperature and were then centrifuged at 3000 rpm/minute for 20 minute. The upper layer of serum was taken and stored at -80°C. The ELISA method was used for the determination of IgG, IgA, IgM, CD4, and CD8 molecules, and the operation and calculation process were carried out in strict accordance with the instructions of the kit. The kits were purchased from Jiangsu Jingmei Technology Co.

### Morphological observations for the duodenal and the jejunal intestine

**Figure 1:**
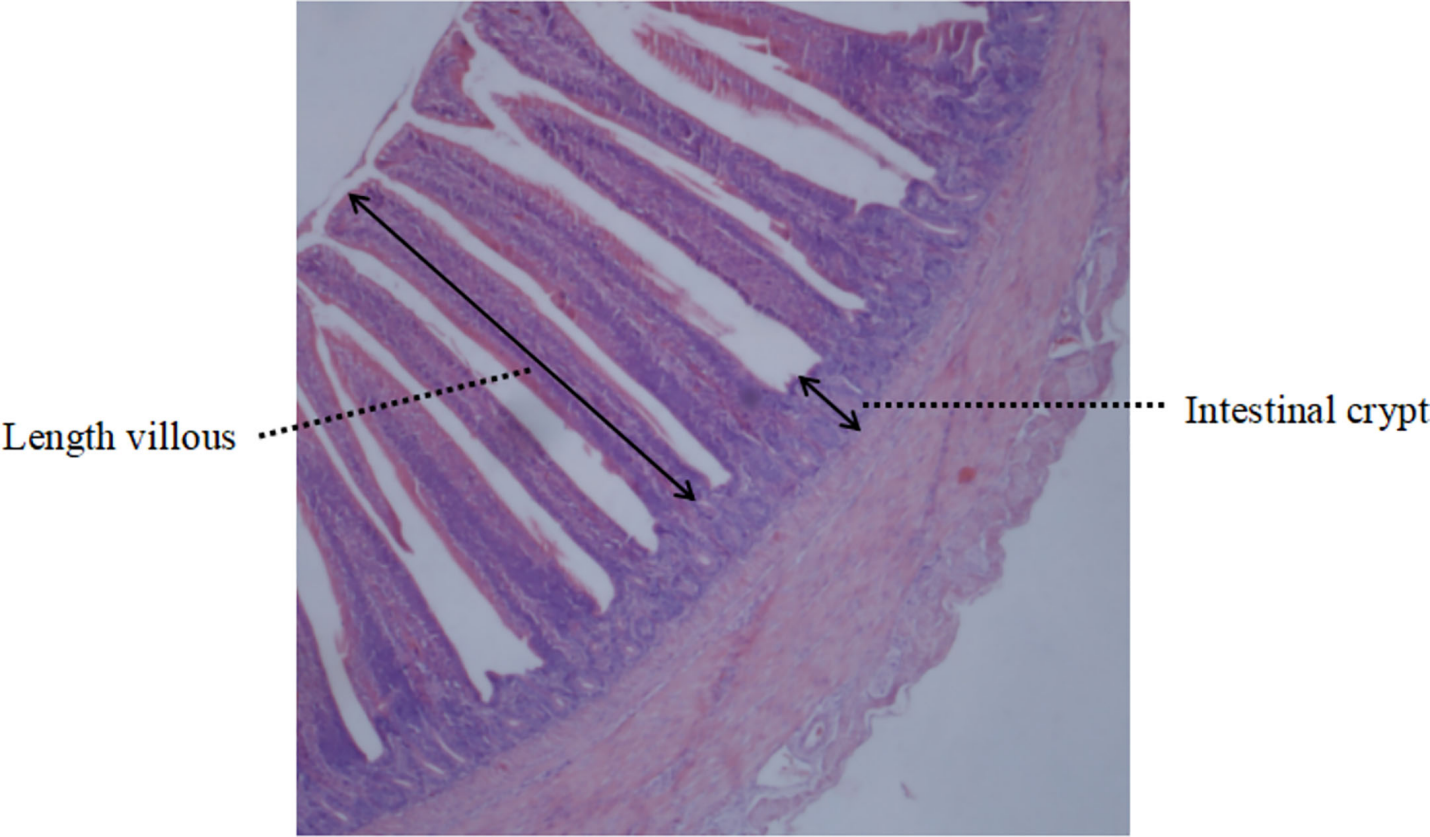
Schematic diagram of intestinal villi.

On day 21 and day 42 respectively, three chickens per replicate were randomly selected and sacrificed after overnight fasting. Duodenal and jejunal intestinal tissue samples were collected, gently rinsed by PBS buffer to remove the intestinal contents, and then placed into 4% paraformaldehyde fixative for fixation. In the Guangxi Institute of Traditional Chinese Medicine, tissue sections were prepared and measured under microscope. Data of duodenal and jejunal villi height and crypt depth were taken and calculated, and the villi height/crypt depth ratio (VCR) were collected. For each sample, two consecutive sections were taken and three different fields of view were randomly selected. The heights of villi (μm) were measured from the tip to the junction of the villi crypt, and crypt depth was defined as the depth of the adjacent villi invagination. The ratios were calculated using the mean value of each group for further comparison (**Figure 1**).

### The expression of jejunal protein mRNA by RT-qPCR method

On day 42, three chickens per replicate were randomly selected and sacrificed after overnight fasting. Jejunal tissue samples were collected and placed in liquid nitrogen. For RNA extraction (performed at 4°C), 100 mg of empty intestine was added to 900 μL of saline and then ground using a tissue grinder, and the samples were subjected to RNA extraction according to the extraction procedure of the RNApure Tissue Kit. Reverse transcription was performed using the HiFiScript cDNA Synthesis Kit. The reaction temperature was 42°C for 15 minutes and the reaction was terminated by increasing the temperature to 85°C for 5 seconds. The UltraSYBR Miture kit was used to quantify the relevant mRNA expression level of the sample. Each reaction contained 1 μL of cDNA template, 0.5 μL of each primer, master mix (12.5 μL), and 10.5 μL ddH2O for a total volume of 25 μL. The fluorescent quantitative PCR (qPCR) was performed using the following procedure: 95°C for 5 min, followed by 40 cycles (95°C for 15 s and 60°C for 1 min). After amplification, melting curve was performed by incubating the reaction at 60°C for 30 s and then increasing the temperature from 60°C to 95°C at a rate of 0.2°C/s. All primer sequences and testing conditions are shown in **Table 2**.

**Table 2:** Fluorescent quantitative PCR.

Gene	Gene Bank Index	Primer seq(5’-3’)	Products/bp	Annealing temperature/°C
CLDN-1	NM_001013611.2	F:ATGACCAGGTGAAGAAGATGC	182	60°C
		R:TGCCCAGCCAATGAAGAG		
OCLN	NM_205128.1	F:TGTCTGTGGGTTCCTCATCG	156	60°C
		R:TTCTTCACCCACTCCTCCACG		
MUC2	XM_040673077.2	F:TCCCTCAAACAAGACCTA	85	60°C
		R:AGAAGTACCACAGCGAAG		
β-actin	NM_205518.2	F:CCCTGTATGCCTCTGGTC	194	60°C
		R:CTCGGCTGTGGTGGTGAA		
ZO-1	XM_046925214.1	F:CGTAGTTCTGGCATTATTCGT	186	60°C
		R:TGGGCACAGCCTCATTCT		
TLR4	XM_040663720.2	F:TGACCACAACCTTGCCTTGA	133	58°C
		R:CACCTGCAAAATGTGACGCT		
TLR2	XM_046915414.1	F:CTGGGAAGTGGATTGTGGA	131	60°C
		R:AAGGCGAAAGTGCGAGAAA		

CLDN-1, Claudin l gene; OCLN, 0ccludin gene; MUC2, Mucin 2 gene; ZO-1, Zonula occludens-l gene; TLR4,Toll-like receptors 4; TLR2, Toll-like receptors 2.

### The analysis of 16S rDNA for intestinal flora population

On day42, three chickens from each testing group were randomly selected and sacrificed after overnight fasting, at which point cecal contents were collected. Total genomic DNA was extracted using the Bacterial Genome DNA extraction Kit (Beijing Kangwei Century Biotechnology Co., Ltd.) following the manufacturer’s instructions. DNA concentration and integrity were measured with Implen NanoPhotometer and agarose gel electrophoresis. Extracted DNA was stored at -20°C until further processing. The extracted DNA was used as a template for PCR amplification of bacterial 16S rRNA genes with the barcoded primers and Takara Ex Taq (Takara). For bacterial diversity analysis, V3-V4 (or V4-V5) variable regions of 16S rRNA genes were amplified with universal primers 343F (5’-TACGGRAGGCAGCAG-3’) and 798R (5’-AGGGTATCTAATCCT-3’) and 907R (5’-CCGTCAATTCMTTTRAGTTT-3’) for V3-V4 regions.

The Amplicon quality was visualized using agarose gel electrophoresis. The PCR products were purified with AMPure XP beads (Agencourt) and amplified for another round of PCR. After being purified with the AMPure XP beads again, the final amplicon was quantified using Qubit dsDNA Assay Kit (Thermo Fisher Scientific,USA). The concentrations were then adjusted for sequencing. Sequencing was performed on an Illumina NovaSeq 6000 with 250 bp paired-end reads. (Illumina Inc., San Diego, CA; OE Biotech Company; Shanghai, China).

The library sequencing and data processing were conducted by OE biotech Co., Ltd. (Shanghai, China). Raw sequencing data were in FASTQ format. Paired-end reads were then preprocessed using Cutadapt software to detect and cut off the adapter. After trimming, paired-end reads were filtered for low quality sequences, denoised, merged, and detected, and the chimera reads were cut off using DADA2 with the default parameters of QIIME2 (2020.11). Finally, the software output the representative reads and the ASV abundance table. The representative read of each ASV was selected using the QIIME2 package. All representative reads were annotated and blasted against Silva database (Version 138) using q2-feature-classifier with the default parameters.

QIIME2 software was used for alpha and beta diversity analysis. The microbial diversity in samples was estimated using the alpha diversity that includes Chao1 index and Shannon index. Then the R package was used to analyze the significant differences between different groups using ANOVA statistical test.

### Statistical analysis

The experimental data were statistically analyzed using SPSS 25.0. Data were presented as means and pooled SEM. The effects of dietary treatment on the measured variables were analyzed by one-way ANOVA followed by Duncan’s multiple comparison test to compare among groups. Differences were considered significant at P < 0.05.

## Results

### *Lactobacillus plantarum* GX17 showed positive effects on growth performance of yellow-feathered broilers

**Figure 2:**
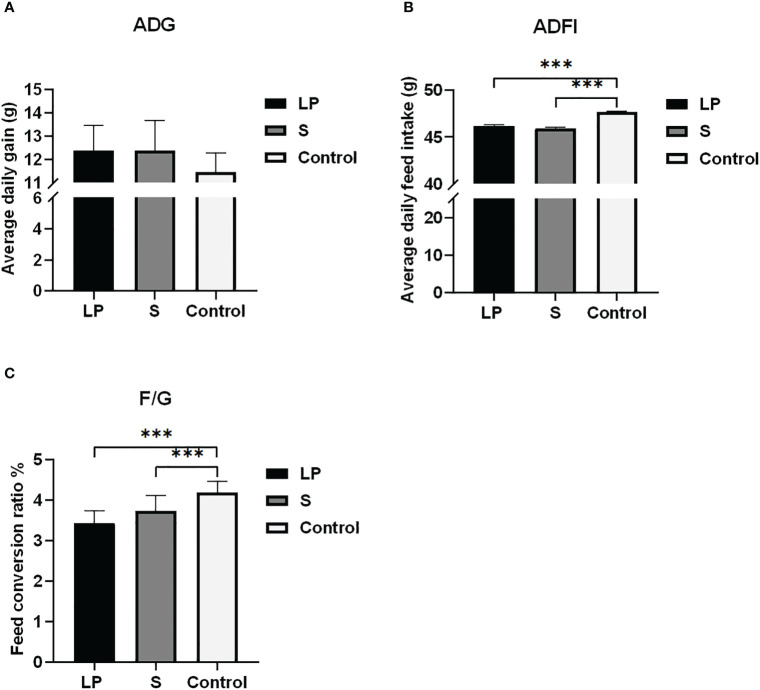
Effects of Lactobacillus plantarum GX17 on growth performance of broilers. **(A)** The average daily gain of the three groups of yellow-feathered broilers on day 42; **(B)** Average daily feed intake of three groups of yellow-feathered broilers on day 42; **(C)** The ratio of feed to gain of yellow-feathered broilers in the three groups (***p<0.001).

As shown in **Figure 2**, although there was no significant difference among the average daily weight gain of yellow-feathered broilers in LP and S groups compared with the Control group, during the whole trial period, the average daily weight gain of yellow-feathered broilers in LP and S groups that were fed probiotics were increased by 0.927 ± 1.33 g and 0.926 ± 1.52 g respectively compared to controls. The average daily feed intake of yellow-feathered broilers in LP and S groups with probiotics added decreased significantly by about 1.497 ± 0.17 g and 1.714 ± 0.13 g (P<0.001). The mean daily feed intake of LP and S groups with probiotics was significantly lower, about 1.497 ± 0.17 g and 1.714 ± 0.13 g (P<0.001). Therefore, after 42 days of probiotic feeding, the feed conversion ratio was significantly lower in both the LP and S groups (P<0.001).

### *Lactobacillus plantarum* GX17 improved immune responses among yellow-feathered broilers

**Figure 3:**
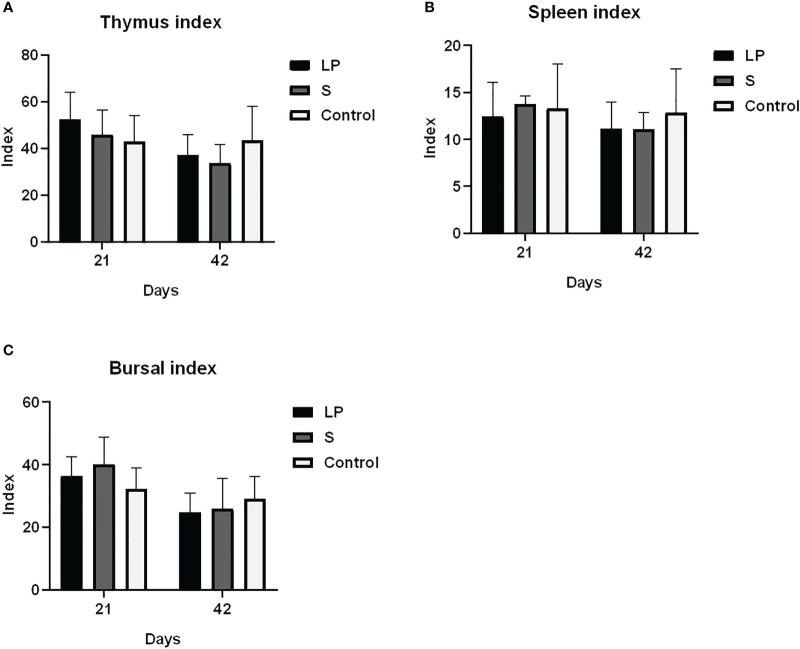
Effects of Lactobacillus plantarum GX17 on immune organ index of yellow-feathered broilers. **(A)** thymus index **(B)** spleen index **(C)** bursae index.

**Figure 4:**
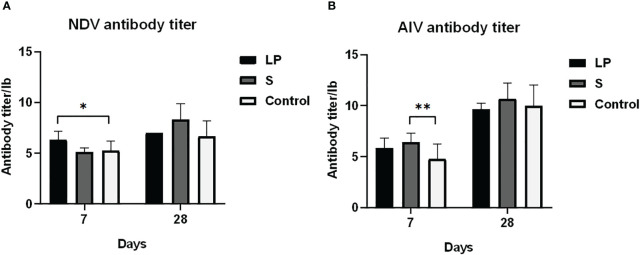
Antibody titer of Lactobacillus plantarum GX17 after immunization against yellow-feathered broilers.(7 and 28 days after immunization) **(A)** Newcastle disease antibody titer **(B)** avian influenza antibody titer (*p<0.05; **p<0.01).

**Figure 5:**
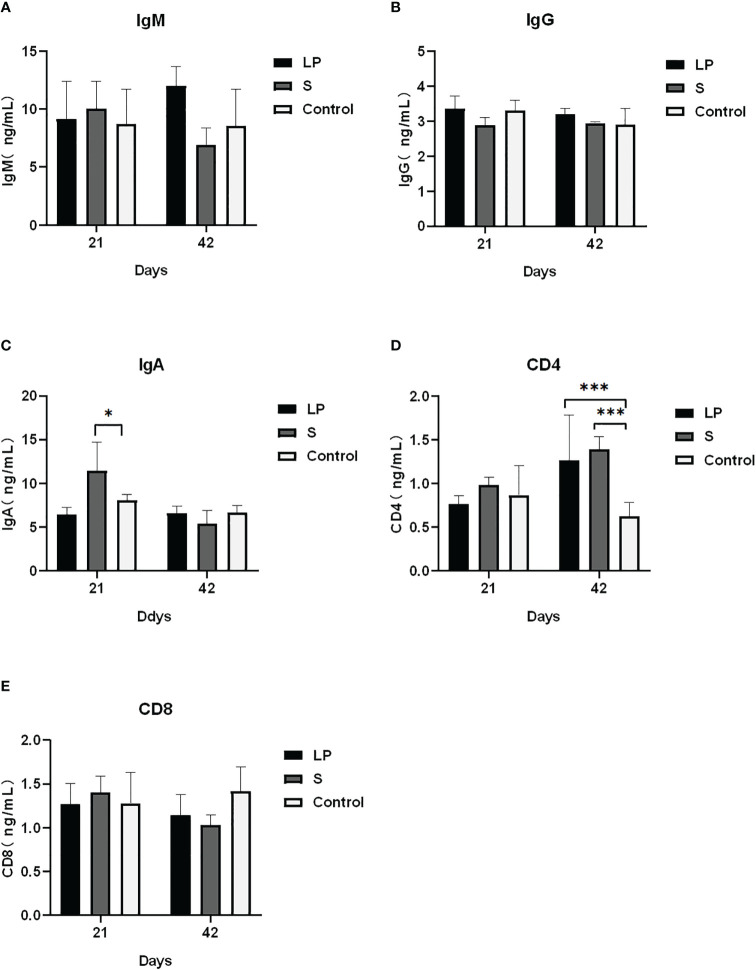
Effect of Lactobacillus plantarum GX17 on serum immunoglobulin content of yellow-feathered broilers. (*p<0.05; ***p<0.001).

As shown in **Figure 3**, *Lactobacillus plantarum* GX17 had no significant effect on the immune organ index of yellow-feathered broilers (P > 0.05). However, it did enhance the antibody potency of yellow-feathered broilers immunized with Newcastle disease vaccine and avian influenza vaccine (**Figure 4**) compared with the Control group. The antibody potency of Newcastle disease was significantly higher in the LP group on the 7th day after the secondary immunization with an increase of about 1.08 potency(P=0.013). The antibody potency of avian influenza was significantly higher in the S group, with an increase of about 1.65 potency (P=0.009). There was no significant difference in the antibody potency of anti-Newcastle disease virus and anti-avian influenza virus between the LP and S groups at Day 28 after the secondary immunization(P>0.05). Feeding *Lactobacillus Plantarum* GX17 also showed positive effects on the immunoglobulin content in the serum of yellow-feathered broilers (**Figure 5**). At Day 21, in both groups which were fed with *Lactobacillus plantarum* GX17 or commercial complex probiotics, the IgM, IgG, CD4, and CD8 contents among groups were not changed (P>0.05); although the S group had a higher IgA content (compare to LP-group: P=0.005; compare to controls: P=0.043). However, at day 42 of feeding, CD4 was significantly higher in the LP and S groups compared to the Control group, with an increase of 102% and 121%, respectively (P<0.001), while IgM, IgA, IgG, and CD8 contents were not significantly changed (P>0.05).

### *Lactobacillus plantarum* GX17 improved the intestinal health of broiler chickens

**Figure 6:**
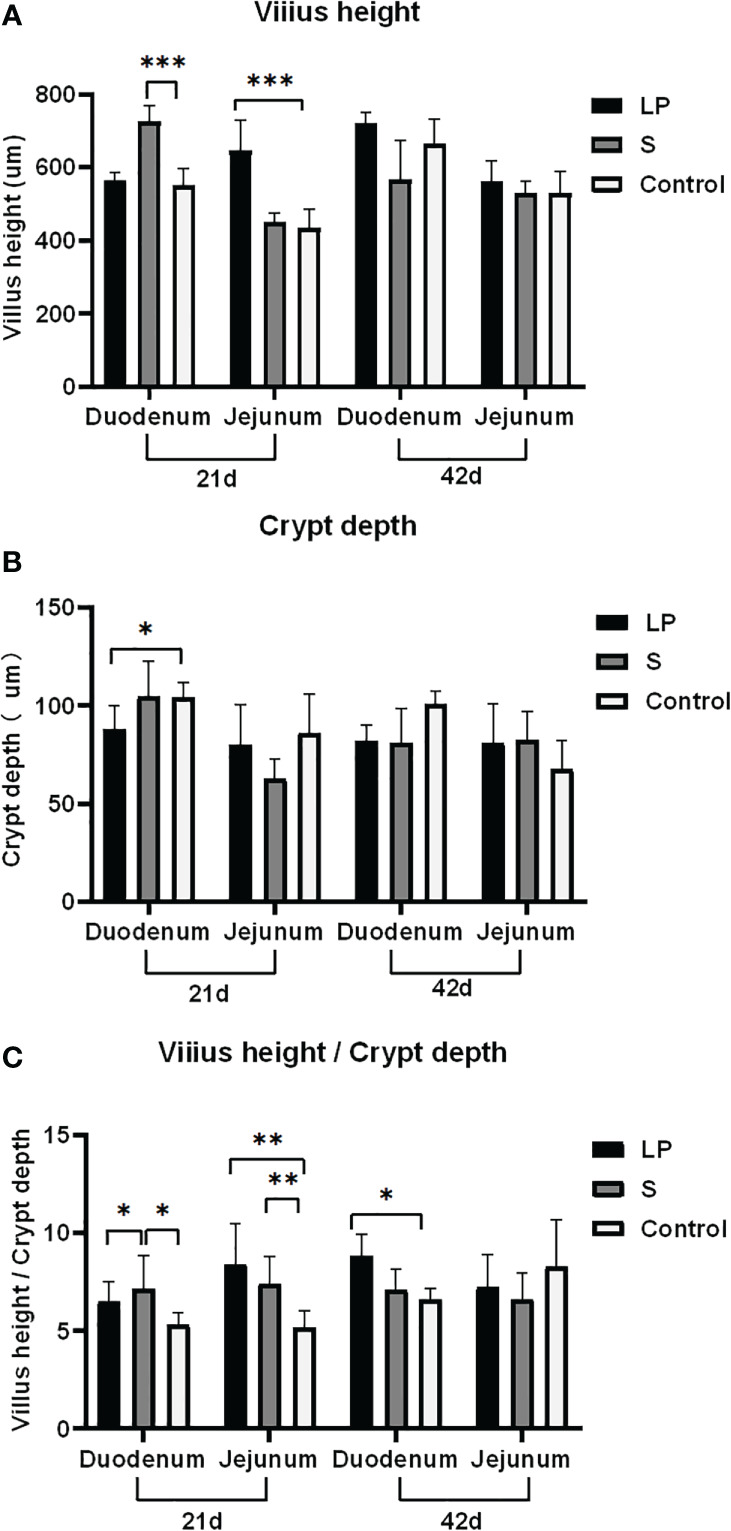
Effect of Lactobacillus plantarum GX17 on intestinal villi of duodenum and jejunum in yellow-feathered broilers.(*p<0.05; **p<0.01; ***p<0.001).

*Lactobacillus plantarum* GX17 promoted the growth of intestinal villi, thereby facilitating the absorption of nutrients. In S group, after feeding probiotics for 21 days, the duodenal VCR ratio increased by 34.7% (P = 0.017) and duodenal crypt depth increased by 31.9% (P <0.001) compared with controls. In LP group, although the duodenal VCR value had a trend of increasing by 22.4% (P = 0.051, >0.05), it was not statistically significant when compared with the control group; the improvement on duodenal crypt depth was not significant either. In jejunum, the S group showed significantly improved VCR value (+43.9%, P=0.006), although their villi length and crypt depth maintained a similar level to the controls. Interestingly, when the length of probiotic treatment was extended to 42 days, no significant differences were observed in villi lengths, crypt depth, or VCR values among all three groups, except the VCR value of LP group increased by 34.1%( P=0.049) (**Figure 6**). This suggests that probiotics may have a stronger facilitation effect for the growth of younger yellow-feathered broilers while the morphological changes were decreased when jejunum and duodenal were matured. However, feeding probiotics for a longer time, particularly GX17, could have advantages in improving the expression of jejunal barrier genes. After feeding Lactobacillus plantarum GX17 for 42 days, the levels of CLDN, MUC2, and TLR2 were significantly higher by about 2.99, 103, and 22.37-fold, respectively, in the LP group compared with controls (P < 0.001). For ocludin, ZO-1, and TLR4, all three showed a trend of increasing compared with the controls, although the result was not statistically significant. When feeding with commercial complex probiotic in group S, the level of CLDN, ocludin, MUC2, TLR2, and TLR4 were increased significantly by 3.04, 4.96, 74.98, 60.30, and 6.57 folds compared to controls (P < 0.001), while only ZO-1 content was not significantly changed.

### Effects of feeding *Lactobacillus plantarum* GX17 on the abundance of intestinal flora among yellow-feathered broilers

**Figure 7:**
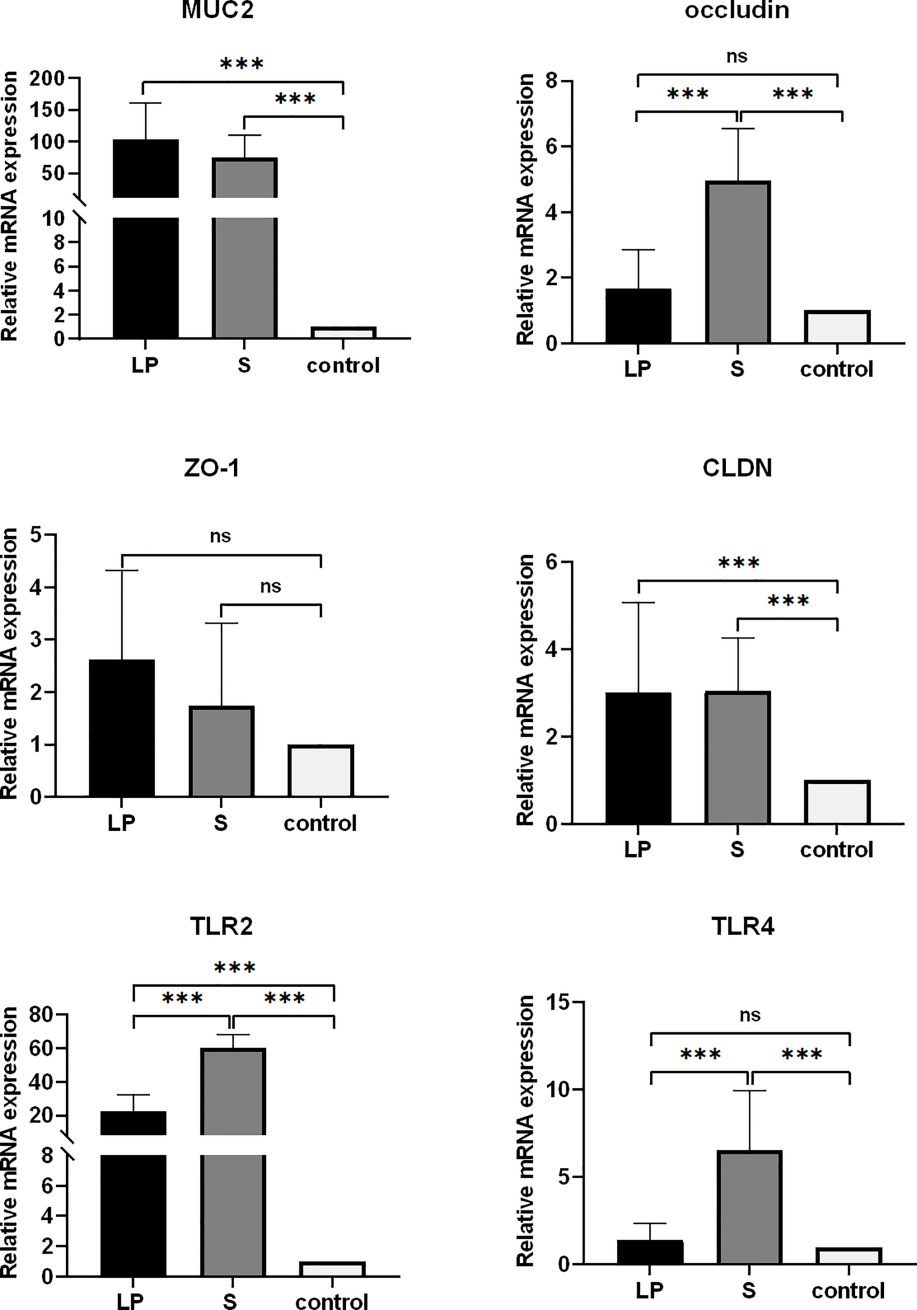
mRNA expression of jejunal barrier related genes (42 days) (all Control values are normalized) CLDN-1: Claudin l gene; OCLN: 0ccludin gene; MUC2: Mucin 2 gene; ZO-1: Zonula occludens-l gene;TLR4:Toll-like receptors 4; TLR2: Toll-like receptors 2 (***p<0.001) ns, no significance.

**Figure 8:**
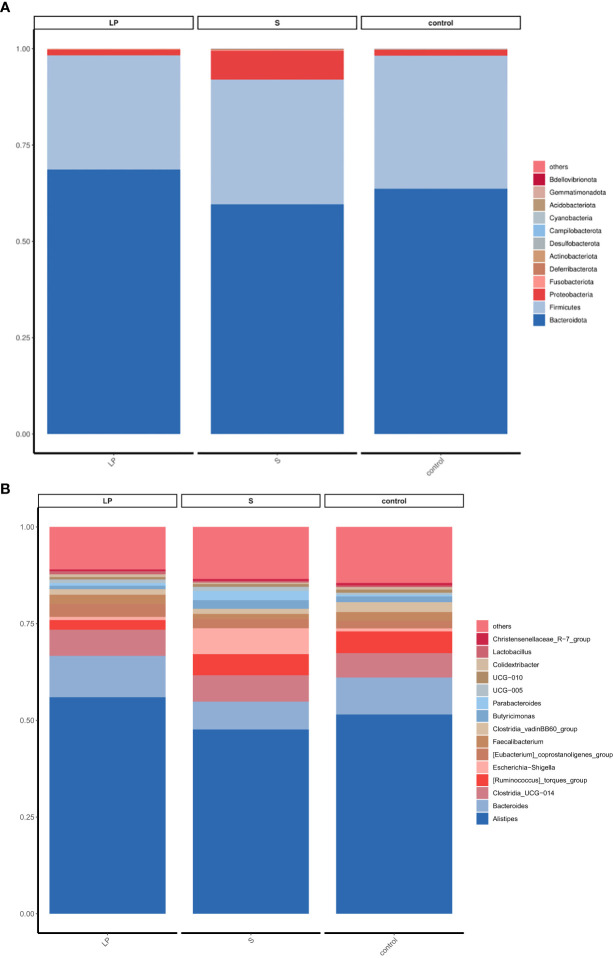
Difference of relative abundance at gate and genus level (42d): **(A)** phylum level; **(B)** genus level.

**Figure 9:**
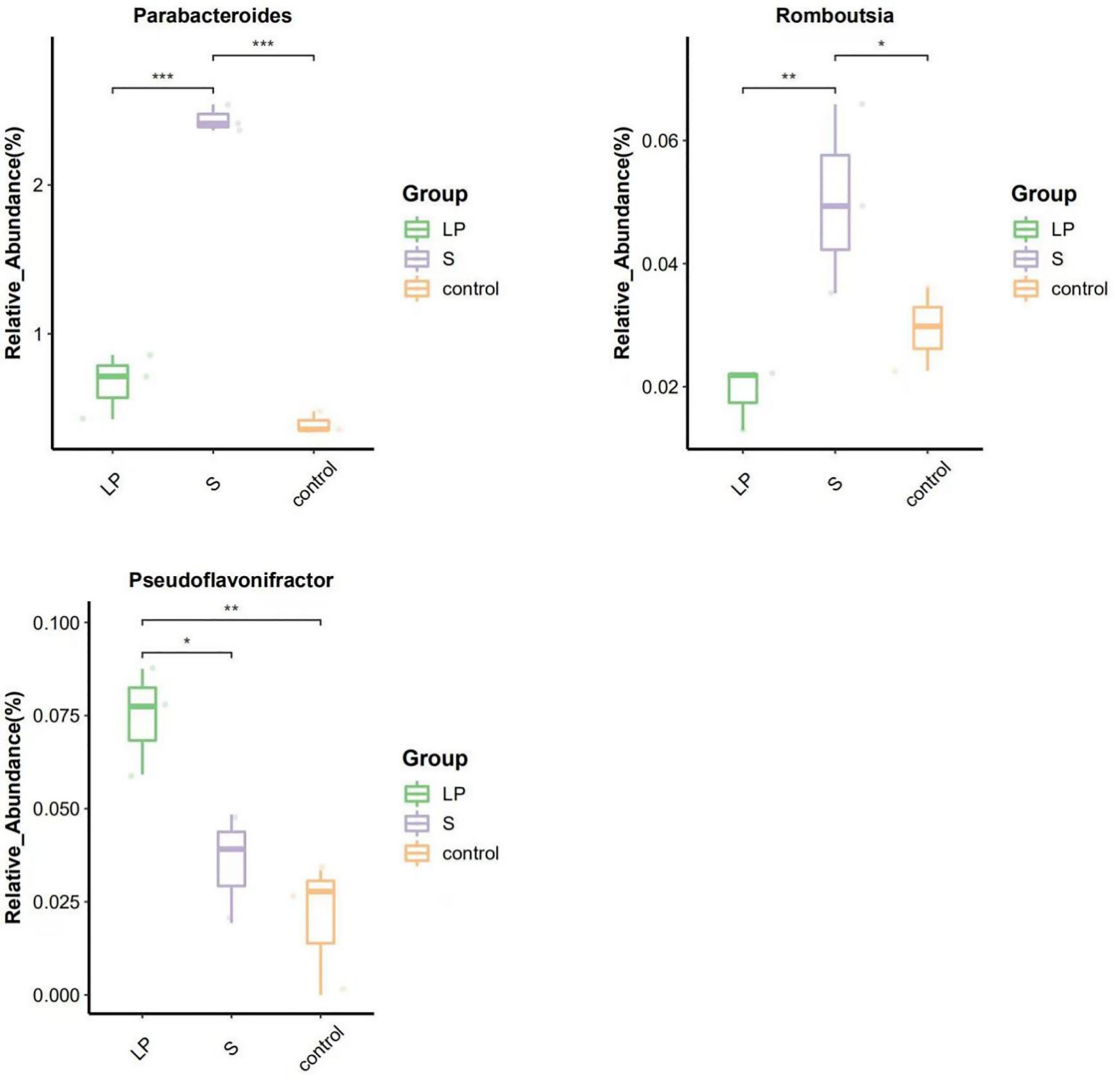
Difference in species abundance Top10 boxplot (42d) at genus level. (^∗^p<0.05; ^∗∗^p<0.01; ^∗∗∗^p<0.001).

To elucidate the underlying mechanisms of the disease-resistant potentiation among yellow-feathered broilers that were fed probiotics, we explored changes of cecum microbiota under diverse testing conditions. We analyzed the diversity of their cecum microbiota at the phylum and genus level to characterize the dynamics of microbial taxonomic distribution. Data showed that at the phylum level, all groups were dominated by *Bacteroidetes*, *Firmicutes*, and *Proteobacteria* (**Figure 7A**). We further compared the bacterial composition of the cecum at the genus level: another set of genus, which include *Alistipes*, *Bacteroides*, *Clostridia*, and *Ruminococcus*, were found to be more dominant (**Figure 7B**). ANOVA analysis showed that at the genus level, the relative abundance of *Parabacteroides* and *Romboutsia* in the S group was 3.66 and 6.17 times higher than the LP group, while these results were 2.64 and 1.70 times higher than controls, respectively. All of these increasing levels were statistically significant, although there was no significant difference between the LP and Control groups. The relative abundance of *Pseudoflavonifractor* in the LP group was 2.10 and 3.66 times higher than that in the S and Control groups, respectively, (**Figures 8**, **9**).

## Discussion

The average daily weight gain, feed intake, and feed-to-weight ratio are important indicators of livestock growth performance. It has been shown that the addition of *Lactobacillus plantarum* B1 to the diet has no significant effect on the performance of broilers in the beginning period, but during the developmental period, the average daily weight gain of broilers treated with *Lactobacillus plantarum* increases significantly and feed conversion ratio is improved (21). Yeo and kim et al. (22) found that the average daily weight gain of broilers fed with probiotics increased significantly in the beginning of the growth period and did not change significantly in the later period. There was no significant change in the later stages. In the present study, it was found that feeding *Lactobacillus plantarum* GX17 and commercial complex probiotic preparations had no significant effect on the growth performance of broilers from 1-42 days of age, which is zconsistent with the findings of Al-Khalaifa (23) et al. However, the feed-to-weight ratio of broilers supplemented with *Lactobacillus plantarum* GX17 and commercial complex probiotics was significantly lower at 1-42 days of age, so it is presumed that the beneficial effect of this probiotic on broilers is the result of long-term feeding, and long-term feeding is required for subsequent production use to achieve improved growth performance. Feeding *Lactobacillus plantarum* GX17 was also observed to reduce feed intake in this study, which is consistent with some studies where supplementation with probiotics reduced feed intake (24), and chen (25) et al. similarly found that the addition of probiotics to broiler diets reduced feed intake. However, some scientists have also observed improved feed intake after supplementation of probiotics in the diet, and probiotics reduced gastric emptying time, which led to more feed intake (26, 27), and instead of reducing the feed to meat ratio, more feed costs were added. In a comprehensive analysis, feeding *Lactobacillus plantarum* GX17 has a tendency to improve the growth performance of broilers, but its period of action may have different effects depending on the strain, content, and feeding route.

Intestinal morphology, including duodenum, jejunum, and ileum villi height, crypt depth, and the ratio of villi height to crypt depth, can reflect the intestinal health of broiler chickens. The increase in the height of small intestinal villi helps to increase the contact area between the small intestine and nutrients and improve the digestion and absorption capacity of the animal organism, while the strong and regular oscillation of intestinal villi can also prevent the colonization of harmful bacteria in the intestine and stabilize the balance of the microbiota (28). The main function of the crypt is to secrete digestive juices, and a decrease in its depth indicates an increase in cell maturity and its ability to secrete digestive juices (29). The increase in villi height and villi height to crypt depth ratio was directly related to the increase in epithelial turnover (30). Arifin et al. found that the feeding of probiotic preparations to broilers resulted in a significant increase in the length and width of small intestinal villi, along with an increase in body weight and a decrease in the feed to weight ratio (31). Song et al. (32) observed that the addition of a probiotic mixture of Bacillus licheniformis, Bacillus subtilis, and *Lactobacillus plantarum* to the diet improved the heat stress-induced shortening of intestinal villi and deepening of the crypt depth in chicks. In this experiment, it was found that feeding *Lactobacillus plantarum* GX17 and the commercial probiotic mixture both significantly increased the length of small intestinal villi and increased the ratio of villi height to crypt depth, which is consistent with the results of many other studies. This indicates that the addition of *Lactobacillus plantarum* GX17 to the diet can improve the growth performance of broilers by improving the intestinal outcome, thereby enhancing the intestinal absorption of nutrients and reducing the feed to meat ratio.

The intestinal mucosal barrier, formed by the intestinal epithelium, is part of the natural immune composition of the intestine, and its intestinal epithelial cells are the first line of defense against foreign pathogens (33). The barrier function of the mucosal surface is mainly in the epithelial cell plasma membrane, which is resistant to many hydrophilic substances due to its lack of specific transport proteins. Barrier function is significantly lost upon direct contact with mucosal antigens or cytotoxic agents, but the cell-to-cell pathway is protected and functions due to the presence of epithelial monomers. And one of the functions is regulated by the apical junction complex, which is a collection of tight junctions and adjacent adherens junctions (AJ) (34). The intercellular tight junctions (TJ) exhibit a complex molecular structure consisting of different types of transmembrane proteins linked to cytoplasmic junctions that contribute to the attachment to neighboring cells, involving interactions between cytoplasmic junction proteins (e.g., occludin ribbons) and integral membrane junction proteins (e.g., occludins and Claudins). These proteins respond to different physiopathological signals through constant regulatory modifications (35).

A variety of probiotics have been found to enhance intestinal barrier function by increasing tight junction-corresponding proteins in the presence or absence of intestinal infection, as well as increasing mucin and heat shock protein production and modulating signaling pathways (33, 36). Certain *Lactobacilli* attenuate barrier disruption by upregulating TJ proteins. *Lactobacillus acidophilus* and *Lactobacillus plantarum* increase the expression of closure proteins in *in vivo* and *in vitro* models, respectively (37, 38). In addition to increasing occludin protein expression, *Lactobacillus plantarum* also induced apical repositioning of ZO-1 and occludin by stimulating TLR2 (39, 40). Izuddin found that the addition of *Lactobacillus plantarum* RG14 to the diet of weaned lambs upregulated the expression of TJP-1, CLDN-1, and CLDN-4 mRNA in the lamb jejunum and improved the integrity of the intestinal barrier (41). It has been shown that *Lactobacillus plantarum* G83 can increase the mRNA expression of ileal Claudin-1, Zo-1, and Occludin-1 by inducing microbiota alterations, thereby improving the intestinal barrier and defending against enterotoxin-producing E. coli K88 (42). Wang et al. (43) found that *Lactobacillus plantarum* ZLP001 could attenuate endotoxin-induced TJ proteins (claudin-1, occludin, and ZO-1) and downregulated the expression and secretion of the pro-inflammatory cytokines IL-6 and IL-8 and tumor necrosis factor α. Zijian et al. found that the reduced expression levels of ZO-1, claudin-1, and occludin caused by a high-fat, high-fructose diet were reduced after *Lactobacillus plantarum* NA136 treatment returned to normal levels (44). In the present study, *Lactobacillus plantarum* GX17 showed the same effect; compared to broilers without probiotic addition, *Lactobacillus plantarum* GX17 upregulated the mRNA expression of CLDN, occludin, and ZO-1 with no significant change in mRNA expression, while CLDN, occludin, and MUC2 mRNA expression was significantly higher in broilers supplemented with commercial complex probiotics (*Lactobacillus*). The difference in their barrier gene expression may be related to the bacterial species and the effect of single versus complex probiotics being not quite the same. MUC2 is the main mucin produced by cuprocytes and is an important component of the mucus layer covering the intestinal epithelium. It has been shown that end-of-diet supplementation with probiotics increased the expression of the MUC2 gene in broiler chickens (45, 46). It has also been found that the downregulation of MUC2 expression induced by endotoxin was antagonized by the addition of Bacillus subtilis (47). In the present study, it was found that the addition of *Lactobacillus plantarum* GX17 to the diet significantly increased the expression of the MUC2 gene in broiler jejunum.

TLRs are a transmembrane protein receptor identified by Lemaitre in his study on Drosophila embryonic development, which was shown by further studies to have a cellular signaling function and a key role in the process of infection and immune response. Xueer Gui et al. (48) found that the relative expression of TLR2, TLR4, TRAF-6, Myd88, and AP-1 mRNAs in the broiler intestine were significantly increased by the addition of complex probiotics to the diet. In this experiment, it was found that the addition of complex probiotics to the diet significantly increased the relative expression of TLR2 and TLR4 mRNAs, and the addition of *Lactobacillus plantarum* GX17 significantly increased the expression of TLR2 mRNA. The beneficial effects of probiotics on intestinal barrier function are mediated, at least in part, by tissue stimulation of TLRs, particularly TLR2, and by sequential changes in the expression and localization of tight junction proteins. There are new studies showing that TLRs interact with intestinal microbiota and epithelial cells in tight junctions. Given their direct interaction with intestinal tight junctions, TLRs play a different and crucial role in the regulation of intestinal tight junctions as a potential probiotic therapeutic target (49). One study found that Caco-2 showed strong apical redistribution of ZO-1 and increased intracellular protein kinase C (PKC) activity after treatment with TLR-2 ligands (50). This may suggest that TLR2 stimulation promotes structural modifications in tight junctions by promoting PKC activity. Thus, insufficient levels of host protective TLR2 ligands may impair barrier function. For example, antimicrobial drug administration may impair barrier function by reducing the luminal population of Gram-positive intestinal commensals such as *Lactobacillus* spp. and *Bifidobacterium* spp., both important sources of TLR2-stimulating ligands (51). Thus, intestinal homeostasis and barrier function require a delicate balance between symbiosis and pathogen-induced TLR activation. This possibility also exists in the present study, where *Lactobacillus plantarum* GX17 and the complex probiotic bacteria enhanced intestinal barrier function by increasing TLR2 expression followed by induction of tight junction protein expression to improve intestinal barrier integrity, but the exact mechanism of action remains to be discovered by further studies.

Gut bacteria play important roles in maintaining the health of the organism which includes vitamin B synthesis, improving digestive and neurological functions, and promoting angiogenesis (52). Because newly hatched chicks lack contact with adult birds, the environmental microbial colonies can be critical for the immunology development of chicks from the hatchery which potentially extends throughout their whole lifespan and can influence the development of the gastrointestinal microbiota (53). In this experiment, probiotics were fed to yellow-feathered broilers from just after hatching to study the effect of probiotics on the gastrointestinal flora of chicks. The microbial community in the gastrointestinal tract of broiler chickens has more than 900 species of bacteria (54). They play crucial roles in digestion, toxin degeneration, pathogen elimination, immune system stimulation, and endocrine activity (55). *Firmicutes*, *Bacteroides*, and *Proteobacteria* are three most common phylums in the chicken cecum, which was confirmed in the present study as the intestinal flora structure was similar among all experimental groups and the main dominant flora were also the three most common phylums mentioned above. The *Parabacteroides* is also the pathogen for inflammatory bowel disease in human beings, and also correlates to carbohydrate metabolism, obesity, and the secretion of short-chain fatty acids (56). It has been found that short-chain fatty acids are important in maintaining healthy intestinal functions, and butyric acid is the main source of energy for colonic epithelial cells (57). Additionally, SCFA could suppress the expression of virulence factors from bacterial pathogens (58). It has also been found that *Parabacteroides* can produce acetate, which attenuates acute pancreatitis in the acetyl heparinase family through reducing neutrophil infiltration (59). The *Romboutsia* is usually found in the intestine of animals and has been associated with their health status. The dramatic decreasing in their abundance in the intestinal mucosa is considered as a microbial indicator representing certain diseases. In healthy animals, the relative abundance of *Romboutsia* is significantly higher than in colorectal cancer tissue. Therefore, the absence of bacteria of this genus is potentially a marker of mucosal pathology (60, 61). Additionally, *Pseudoflavonifractor* is a common cecum-colonizing bacterium with proteins derived from class IV alcohol dehydrogenases that influence the final butyrate production pathway (62). In this study, the relative abundance of *Parabacteroides* and *Romboutsia* were both significantly higher in the intestine of yellow-feathered broilers fed with commercial complex probiotics, and the relative abundance of *Pseudoflavonifractor* was significantly higher in the intestine of yellow-feathered broilers fed with *Lactobacillus plantarum* GX17. These data suggest that commercial complex probiotics and *Lactobacillus plantarum* GX17 are both able to maintain the cecum health of yellow-feathered broilers by promoting the proliferation of specific bacterial.

It has been reported that the weight of the thymus, spleen, and bursa reflect the immune status of chicks, and the increase in the absolute and relative mass of these organs indicates the enhancement of cellular and humoral immunity (63). Although the application of immune enhancers can improve the immune functions of poultry while enhancing their resistance to infectious diseases, most immune enhancers are chemically synthesized with significant side effects, drug residue issues, and other food safety risks. Previous studies have demonstrated that feeding broilers probiotics could significantly increase their immune organ index, indicating an enhancement of their immune function (64). Although some other studies held the opposite result that the growth of broiler spleen was inhibited after 21 days of feeding with commercial probiotic compounds (65). Feeding *Lactobacillus plantarum* GX17 neither stimulated nor inhibited the growth of immune organs, indicating that broilers have a strong adaptation ability to the colonization of the GX17 strain. Since the GX17 strain produces no adverse immune effect on broilers, it could be a potential feed additive fit for daily application in young chicks. Based on our data, the supplementation of probiotics could improve the intestinal barrier function and enhance the intestinal immunity, which leads to the increase of antibody level by improving the intestinal tract, and the increase of beneficial microorganisms may also help to enhance the effect after vaccination.

The level of immune antibodies is a common indicator of immune function, among which the level of Newcastle disease and avian influenza antibodies can better reflect the specific humoral immune response of chickens. Produced by bone marrow-derived B lymphocytes, lgG, IgM, and lgA are immunoglobulins with antibacterial, antiviral, and antitoxin effects (66), which are the main effector molecules of humoral immunity and are essential in humoral immunity. The immune capacity of the body can be reflected by the level of immunoglobulins in the serum, which is also an important indicator to evaluate the changes in the immune function of animals. It has been shown that probiotics not only promote the development of the immune organs of the organism, but also have a modulatory effect on vaccines and improve the specific humoral immune function of the organism (67). Talebi et al. (68) reported that the administration of probiotics in conjunction with vaccination produced a better antibody response. In the present experiment, feeding *Lactobacillus plantarum* GX17 significantly increased Newcastle disease antibody potency at day 7 post-immunization and the results of the study are consistent with the data from Talazadeh et al. (69) that show a significant increase in antibody titers against Newcastle disease vaccine after feeding probiotics, but no significant change in antibody titers against avian influenza virus vaccine. This may be due to the fact that feeding probiotics could enhance the antibody level of the organism against some specific antigens. Meanwhile, Isolauri et al. (70) found that feeding *Lactobacillus* casei during rotavirus vaccination increases the amount of IgM and IgA antibody-secreting cells of the organism, therefore increasing antibody production. In the present study, yellow-feathered broilers fed *Lactobacillus plantarum* GX17 had significantly higher levels of CD4 molecules in the serum at the 28th and 42nd day after the second vaccination compared to the control group (vaccinated only). It has been reported that probiotics play a key role in stimulating the development of the immune system during their initial entry and colonization of the intestine, with a wide range of immune-enhancing functions such as activation of immune cells, increase in number and promotion in maturation of T/B cells, enhancing immune recognition, inducing the expression of relevant immune factors, and activating the whole immune system of the host (71, 72). *Lactobacillus* has been shown to improve humoral immunity response of chickens to live Newcastle disease vaccine, particularly during heat shock period (73). In a trial with weanling rabbits, it was observed that feeding *Lactobacillus* licheniformis significantly improved humoral immunity responses and vaccination following probiotic feeding and was superior to vaccination alone (74). In the present experiment it was demonstrated that *Lactobacillus plantarum* GX17 used synergistically with the vaccine showed better enhancement of both cellular and humoral immunity than the vaccine alone. It was strongly demonstrated that feeding *Lactobacillus plantarum* GX17 could improve the humoral immune function of broiler chickens and enhance the immune effect of Newcastle disease and avian influenza vaccines at the same time.

## Data availability statement

The original contributions presented in the study are publicly available. This data can be found in the NCBI repository, accession number: PRJNA950182.

## Ethics statement

The animal study was reviewed and approved by The Institutional Animal Experimental Ethical Inspection Form of Guangxi Veterinary Research Institute, Nanning, China.

## Author contributions

YYY: Conceptualization, methodology, investigation, writing—original draft preparation, and funding acquisition. YYL: methodology, software, formal analysis, data curation, and writing—original draft preparation. JL: methodology, software, formal analysis, data curation, and writing—original draft preparation. ZP: formal analysis, writing—review and editing, and investigation. LPW: methodology, writing—original draft preparation, and resources. YS: methodology, investigation, writing—review, and editing. HYP: methodology and investigation. YZT: methodology and investigation. CTL: software. HLB: software. CXM: writing—review and editing. YG: writing—review and editing. TCW: Conceptualization, formal analysis, resources, supervision, and project administration. HP: Conceptualization, formal analysis, resources, supervision, project administration, and funding acquisition. All authors contributed to the article and approved the submitted version. All authors have read and agreed to the published version of the manuscript.
